# Evaluating the Construct Validity and Sensitivity to Change of the Klenico Depression Domain in Psychotherapeutic Inpatient Care: Instrument Validation Study

**DOI:** 10.2196/50504

**Published:** 2025-07-24

**Authors:** Stefan Reutimann, Jasmin Steiner, Noah Hübscher, Ulrich Voderholzer, Adrian Meule, Mareike Augsburger

**Affiliations:** 1 University of Zurich Start-Up Klenico Health AG Zürich Switzerland; 2 Department of Health Sciences and Technology Swiss Federal Institute of Technology Zurich Switzerland; 3 Department of Psychiatry and Psychotherapy Ludwig Maximilian University of Munich Munich Germany; 4 Schoen Clinic Roseneck Prien am Chiemsee Germany; 5 Department of Psychology University of Regensburg Regensburg Germany

**Keywords:** depression diagnosis, Klenico system, Klenico Depression Domain, sensitivity to change, psychometric properties, construct validity, mental health assessment, clinical outcome monitoring

## Abstract

**Background:**

The accurate diagnosis of mental disorders, such as depression, requires comprehensive, valid, and reliable tools to ensure evidence-based treatments and effective outcome monitoring. Existing diagnostic practices often lack standardization, leading to missed comorbidities and variable diagnostic accuracy. The Klenico system is an innovative, web-based diagnostic tool that integrates patient self-reports with clinical validations by mental health professionals. This system covers a broad spectrum of mental disorders, including depression.

**Objective:**

This research aimed to evaluate the psychometric properties of the Klenico Depression Domain (KDD), the component of the Klenico system that measures depressive symptomatology, in a real-world clinical setting. Specifically, the evaluation focused on the assessment of its construct validity, internal consistency, and sensitivity to change in symptom severity.

**Methods:**

Anonymized data from 496 inpatients with mental disorders collected between 2019 and 2022 were analyzed. Patients completed the KDD alongside parts of the Patient Health Questionnaire (PHQ), Beck Depression Inventory (BDI-II), and Satisfaction With Life Scale (SWLS) at both admission and discharge. Internal consistency was measured using Cronbach α. Exploratory factor analysis was conducted to examine the factor structure. Construct validity was assessed via Pearson correlations with PHQ-9 and BDI-II, while divergent validity was tested against the PHQ Somatic Symptoms Scale (PHQ-15), PHQ–Generalized Anxiety Disorder-7, and SWLS. Sensitivity to change was evaluated using paired 1-tailed *t* tests, effect sizes, and repeated measures correlations.

**Results:**

The KDD demonstrated excellent internal consistency (Cronbach α=0.91 at admission and 0.93 at discharge). Factor analysis revealed a 7-factor structure encompassing dimensions like “inadequacy,” “anhedonia,” and “self-hatred,” aligning with core depressive symptoms outlined in the *International Statistical Classification of Diseases, Tenth Revision*. The correlations with the convergent questionnaires PHQ-9 (*r*=0.68; *P*<.001) and BDI-II (*r*=0.70; *P*<.001) were high. While the KDD showed a moderate correlation with the divergent PHQ-15 (*r*=0.35; *P*<.001), it was more strongly associated with the divergent SWLS (*r*=–0.51; *P*<.001) and Generalized Anxiety Disorder-7 (*r*=0.51; *P*<.001). Sensitivity to change was high, with significant reductions in KDD scores for patients with improved symptoms (*t*_27_=5.36, *P*<.001; Cohen *d*=0.79) and high repeated measures correlation with both the BDI-II (*r*=0.61; *P*<.001) and the PHQ-9 (*r*=0.59; *P*<.001).

**Conclusions:**

The KDD shows promise as a reliable and valid instrument for diagnosing depression and monitoring treatment outcomes in psychotherapeutic settings. Its alignment with *International Statistical Classification of Diseases, Tenth Revision* diagnostic criteria and sensitivity to symptom change underlines its potential utility. These findings highlight the Klenico system’s potential to enhance clinical diagnostics by addressing current gaps in mental health care, thus improving diagnostic accuracy and consistency. Further research is recommended to validate its performance across different populations and settings.

## Introduction

Mental health disorders represent a broad spectrum of conditions with significant impacts on individuals’ lives globally [1]. These disorders impair both personal and social functioning [2] and pose substantial challenges to public health systems [3]. For example, depression affects a significant portion of the European population, with a lifetime prevalence of 12% and an annual prevalence of about 7% [4,5], imposing considerable economic burdens of roughly €3000 (≈US $3549) per patient per year [6]. This highlights the critical need for qualitative diagnosis and treatment strategies across the spectrum of mental disorders. However, the diagnosis of mental disorders remains challenging due to common comorbidities among different disorders [7,8] and their complex structures. For instance, depression is a highly heterogeneous disorder characterized by a wide variety of different presentations and a broad range of symptoms [9].

In current clinical practice, the quality of diagnostic procedures is often compromised by a lack of standardization and comprehensiveness. Health professionals predominantly tend to rely on unstructured, open interviews for diagnosis and classification [10-12], which compromises validity and reliability [13,14], thereby impeding evidence-based diagnoses [15]. Standardized tools like structured interviews are rarely used in real settings, as they are considered too time-consuming [10,11]. Alongside structured interviews, self-report questionnaires have emerged as an additional standardized method of assessment, seeing more frequent application in routine diagnostic procedures. Although these questionnaires are valid and time-saving tools when implemented for their intended fields of use, such as screening, severity assessment, and course measurement, they do generally not cover the whole spectrum of symptoms [16]. Focusing solely on one particular disorder domain, such as depression, using a disorder-specific questionnaire may lead to missing critical comorbidities. Moreover, self-report questionnaires lack consistency, as they measure a wide variety of different constructs and show inhomogeneity in the represented diagnostic criteria. For instance, the most common depression self-report questionnaires revealed only a 39% similarity in their covered symptoms [17]. Therefore, self-assessments and their cutoff scores alone should not be relied on for diagnosis [18]. It is necessary to obtain further clarifications on comorbidities and symptom duration through structured diagnostic interviews [19]. Alongside initial diagnostics, there is a significant lack of standardized measurement techniques for assessing outcomes [20-22]. However, this step is indispensable for the precise determination of treatment success [23].

The described lack of quality standardization and comprehensiveness in clinical practice causes mental disorders to often be unrecognized or misdiagnosed in different settings [15,24-28]. For instance, studies comparing routine diagnoses made by unstructured, unstandardized diagnostic procedures with standardized diagnoses made by semistructured interviews—the gold standard in diagnostics—among psychiatric inpatients and outpatients found that depression was correctly identified in only half of the cases. The agreement for most other disorders was also limited or poor [14,15,29]. Additionally, comorbidity is often missed in routine diagnostics [30]; for example, the frequent comorbidity between depression and anxiety was detected in fewer than half of the cases using unstandardized routine diagnostics [29].

These inadequate diagnostic practices, among other possible reasons such as poor access to therapy [31], can lead to ineffective therapeutic interventions. This situation may culminate in unwarranted patient distress and increased demands on health care resources [32]. This is reflected by the fact that only 60% of diagnosed depression patients are provided with evidence-based therapy according to guidelines in primary care in Germany [33], and only one-third of mental health treatments in Spain meet minimal adequacy criteria in both specialty and general medical care [34], with similar findings in other countries and for other mental disorders [35-37].

To enable efficient and adequate treatments, diagnostic procedures need to assess the entire symptomatology of mental disorders in a standardized way, allow conclusions about individual symptoms, and be able to comprehensively record potential comorbidities by querying all relevant disorders. In addition, it is important to adequately measure the course of symptoms to evaluate the success of treatments [23]. However, they also have to be practical, efficient, user-friendly, and feasible in clinical practice due to time constraints [38].

Digital technologies offer a promising solution for the described situation. These tools not only facilitate the diagnostic process but also offer the capability for self-administered assessments, thereby reducing the burden on healthcare professionals and enhancing the accessibility of diagnostic services by overcoming conventional barriers. Through the application of adaptive testing, a wide array of symptoms can be efficiently evaluated, optimizing both time and comprehensiveness. Furthermore, digital tools can empower professionals in standardized diagnostics by supporting their decision-making processes.

To this end, the Klenico system was developed as a comprehensive, web-based diagnostic tool that covers the following mental disorder domains: anxiety disorders, disorders of adult personality and behavior, eating disorders, mental and behavioral disorders due to psychoactive substance use, depression, mania, obsessive-compulsive disorder, reaction to severe stress and adjustment disorders, and psychotic disorders. Furthermore, the system includes aspects of autism spectrum disorder, attention-deficit/hyperactivity disorder, and dementia. Its items are directly derived from the diagnostic criteria of the *International Statistical Classification of Diseases, Tenth Revision* (*ICD-10*) [39]. The Klenico Depression Domain (KDD) investigated in this research covers all *ICD-10* depressive disorders under F32 and F33, as well as dysthymia (F34.1), providing a comprehensive representation of depressive symptomatology.

**Figure 1:**
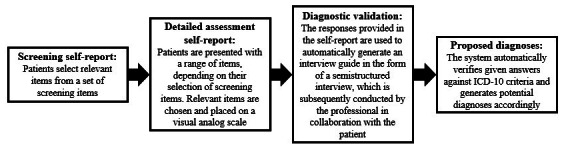
Flowchart depicting the entire process of the Klenico system. ICD-10: International Statistical Classification of Diseases, Tenth Revision.

More specifically, Klenico consists of a web-based, adaptive self-report module with a total of 496 items, including a preceding screening as well as subsequent clinical validation in the form of a diagnostic interview conducted by mental health professionals. The detailed process of the Klenico system is illustrated in Figure 1. Lustig [40] provides more details on the development of the Klenico system. For clinical validation, the software creates an interactive interview guide based on responses to self-reports. This allows the professional to assess the symptomatology reported by the patient and reevaluate it externally through their expertise by asking questions and making observations. The combination of self-report and external assessment is crucial for standardized, multimodal diagnostics and serves as the ideal procedure. This integration facilitates efficient diagnoses of depression and other disorders that are easily implemented in everyday clinical practice and can relieve the burden on professionals while still maintaining a standardized and comprehensive diagnostic approach. Based on the results of the clinical validation, the system generates final automated suggestions for the most likely diagnoses, ensuring strict adherence to the diagnostic criteria outlined in the *ICD-10*. Moreover, the manifestations of the individual symptoms are clearly visually presented on a so-called symptom map, which enables targeted assessment at the symptom level and reveals important comorbidities. Furthermore, the assessments can be repeatedly performed throughout the course of treatment, whereby symptom change is made visible, thereby enabling treatment outcomes to be monitored.

The validity of the Klenico self-report module was preliminarily confirmed on an outpatient sample in a previous study. The study showed that the diagnostic results from the Klenico self-report module aligned well with those obtained from the Structured Clinical Interview for *Diagnostic and Statistical Manual of Mental Disorders* (*DSM*)*, Fourth Edition*, thus demonstrating its criterion validity. Furthermore, preliminary indications of its convergent and divergent validity were also established through the comparison with common self-report questionnaires [41]. The purpose of this assessment was to confirm these findings and further validate the Klenico system by laying the focus on its depression domain (KDD). Research was focused on the KDD’s reliability, construct validity, and sensitivity to change by considering the measured severity of the symptomatology. This is of substantial importance for ensuring the quality of the diagnostic tool and thus enabling valid, adequate, and standardized diagnostics of depression and mental disorders in general.

## Methods

### Procedure

The data used in the present research were collected between 2019 and 2022 in a German psychotherapeutic inpatient clinic, which specializes in treating patients from a range of diagnostic groups. The clinic’s standard diagnostic procedure was administered to all patients, which provided the foundation for the data used in this study. The Klenico system was added provisionally to these standard procedures as an extension to the usual care.

Upon admission, all inpatients received their routine diagnoses from the clinic based on an initial interview and received routine clinical self-report questionnaires (Beck Depression Inventory [BDI-II], Patient Health Questionnaire [PHQ]–9, PHQ-15, PHQ–Generalized Anxiety Disorder-7 [GAD-7], and Satisfaction With Life Scale [SWLS]) to further clarify their symptoms. Afterward, all patients were informed about the procedure and were asked to sign informed consent to allow their routine data and data from the Klenico assessment to be used for scientific studies and publications. Patients then received access to the Klenico self-report module.

Patients then completed the Klenico self-report module on a device of their choice (computer or tablet) and the clinics’ routine self-report questionnaires within the first 2 weeks of their stay. The German version of the Klenico system was used. After completion, mental health professionals conducted the Klenico clinical validation module together with the patient in their next session. At discharge, the procedure was repeated while patients again were asked to answer the clinic’s routine self-questionnaires and the Klenico self-report module. Additionally, the Klenico clinical validation module was performed again. No diagnoses were made in the data collection based on the Klenico system.

Mental health professionals consisted of licensed psychotherapists, psychotherapists in training under supervision (with a minimum of 1 year of professional experience), and cotherapists. Professionals received an introductory session and a demonstration of the use of the Klenico system prior to their initial diagnostic validation. They were also provided with instructions on how to interpret and elucidate the results. The data were fully anonymized.

### Measures

#### BDI-II Questionnaire

The BDI-II was part of the clinic’s routine diagnostic procedure to evaluate depressive symptom severity and to serve as a measure of progress at discharge. The BDI-II [42] evaluates the severity of symptoms of depression in the past 2 weeks. It includes 21 items that are summed up to a maximum total score of 63. The BDI-II has good validity and reliability [43], including test-retest reliability [44], and is sensitive to symptom change [45]. The sum score was calculated. Cronbach α coefficient was 0.91 in this analysis.

#### PHQ-D Questionnaire

The PHQ-D [46] is a self-report instrument for recording mental disorders. The questionnaire demonstrates construct validity based on the number of doctor visits, psychosocial impairment, and incapacity to work, as well as excellent criterion validity (85% sensitivity and 70% specificity) when compared with Structured Clinical Interview for *DSM-IV, Axis I* diagnoses. Both professionals and patients show high acceptance of the PHQ-D, with a satisfaction rate greater than 90% [47]. The short form (option C according to the manual), which screens for depressive (PHQ-9), generalized anxiety (GAD-7), and somatoform (PHQ-15) symptoms, was used in the data collection as part of the clinic’s routine diagnostic procedure at admission and discharge to evaluate symptom severity and to measure progress. The PHQ-9 has good test-retest reliability [48] as well as sensitivity to symptom change [49]. Sum scores of the PHQ-9 (maximum score=27), PHQ-15 (maximum score=30), and GAD-7 (maximum score=21) were calculated. Cronbach α coefficients for the PHQ-9, PHQ-15, and GAD-7 were 0.84, 0.78, and 0.83, respectively.

#### SWLS Questionnaire

The SWLS was part of the clinic’s routine diagnostic procedure upon admission and is a 5-item questionnaire for measuring life satisfaction [50]. Items are summed up to a maximum score of 35, indicating extreme satisfaction. The SWLS has factorial validity as well as reasonable convergent and divergent validity indicated through correlations with other indicators of quality of life [51,52]. The sum score was calculated. Cronbach α coefficient was 0.86.

#### Klenico (Including the KDD)

The Klenico system is a web-based software designed to assist professionals in diagnosing mental disorders. It comprises 2 modules: a self-report followed by clinical validation. The general process of the Klenico system is depicted in Figure 1. Initially, the self-report module presents patients with 55 screening items, from which they select the relevant ones. Depending on the screening items selected, up to 496 items may then be adaptively presented to the patient for a more detailed assessment. The patient again selects the applicable ones and rates each previously selected item by dragging and dropping it on a visual analog scale (ranging from 0=low to 100=high) to assess symptom severity. Upon completion by the patient, the process transitions to the professional. The results from the self-report are graphically displayed on a symptom map, and an interview guide is generated for use in the subsequent clinical validation. This clinical validation involves a semistructured interview where the professional assesses the self-report from an external perspective. This includes posing questions based on the self-reported items, now reformulated for external inquiry, alongside additional external assessment items. These items are rated on a 4-point Likert scale. The system then compares the responses with *ICD-10* diagnostic criteria and provides the professional with suggested diagnoses.

In the data collection used for this assessment, the Klenico study version was used, in which all available 496 items are presented in the detailed assessment of the self-report, regardless of the previously selected screening items.

The KDD assessed in this assessment consists of a total of 31 items, with 29 items included in the self-report module. The clinical validation module reiterates these 29 items as external interview questions, with 2 additional observational items (on psychomotoric agitation and retardation) made by the professional. Means of the KDD for the clinical validation module were calculated where possible. For patients who solely answered the self-report module, means for the self-report were calculated consequently. Cronbach α coefficient was 0.91. Item names, means and SDs of the different KDD items, as well as an exemplary selection of the item formulations, are presented in Multimedia Appendix 1.

### Sample

Fully anonymized data from 496 adult inpatients with mental disorders were used (337 female patients and 159 male patients). Data from discharge were available for 59 patients. Data from the Klenico clinical validation module were available for 445 patients at admission and 42 patients at discharge. The mean age was 38.9 (SD 15) years, and the mean duration of stay was 70.2 (SD 38.6) days. A total of 391 patients received a diagnosis of depression. The frequencies of the different depression diagnoses are reported in Table 1 (for a full overview of all given diagnoses, see Multimedia Appendices 2 and 3 [somatic diagnoses]).

**Table 1:** Overview of the given depression diagnosis with corresponding International Statistical Classification of Diseases, Tenth Revision (ICD-10) codes. The diagnoses were established through the initial interview as part of the routine diagnostic procedures followed at the clinic. Listed frequencies are relative to the number of total given depression diagnoses.

Depression diagnosis	*ICD-10* code	Frequency (n=391), n (%)
Mild depressive episode	F32.0	4 (1)
Moderate depressive episode	F32.1	75 (19.2)
Severe depressive episode without psychotic symptoms	F32.2	41 (10.5)
Severe depressive episode with psychotic symptoms	F32.3	1 (0.3)
Recurrent depressive disorder, current episode mild	F33.0	7 (1.8)
Recurrent depressive disorder, current episode moderate	F33.1	135 (34.5)
Recurrent depressive disorder, current episode severe without psychotic symptoms	F33.2	115 (29.4)
Recurrent depressive disorder, current episode severe with psychotic symptoms	F33.3	2 (0.5)
Recurrent depressive disorder, currently in remission	F33.4	1 (0.3)
Dysthymia	F34.1	10 (2.6)

### Statistical Analysis

Data analysis was performed using RStudio software (version 2021.9.1.372; Posit, PBC). *P* values <.05 were considered statistically significant.

Exploratory factor analyses (EFA) were conducted to explore the underlying factor structure of the KDD using the *lavaan*, *SEMtools*, and *psych* packages. Kaiser‒Meyer‒Olkin (KMO) and Bartlett tests were used to test the adequacy of the factor analysis using the functions *cortest.bartlett* and *KMO*. Parallel analysis and fit estimation were used to identify the optimal number of factors using the functions *efaUnrotate* and *fa.parallel*. A maximum likelihood estimator with robust standard errors was applied. Oblique rotation was applied by using the *oblqRotate* function (the R code for the EFA can be found in Multimedia Appendix 4). The model fit was considered adequate when the comparative fit index >0.90, Tucker‒Lewis coefficient >0.90, standardized root mean square residual <0.10, and root mean square error of approximation <0.08 [53]. Items were assigned to a specific factor if they demonstrated a loading greater than 0.30 on that factor.

Convergent validity for the KDD was tested by performing Pearson correlation analysis with both the PHQ-9 and BDI-II, while divergent validity was assessed by comparison with the PHQ-15, GAD-7, and SWLS. An r value from >0.1 to <0.3 was considered to indicate a small effect, from ≥0.3 to <0.5 was considered to indicate a medium effect, and ≥0.5 was considered to indicate a large effect [54]. Bonferroni-adjustment was performed to correct for multiple testing.

Sensitivity to change of the KDD was examined by categorizing each patient into groups of either improved or unchanged symptoms based on changes in the PHQ-9 or BDI-II sum scores. KDD means for admission and discharge were compared for both improved and unchanged symptom groups by paired 2-tailed *t* tests. Effect sizes and standardized response means were calculated. Additionally, repeated measures correlations between the KDD and the BDI-II and between the KDD and the PHQ-9 were calculated using the *rmcorr* package [55]. Bonferroni adjustment was performed to correct for multiple testing.

A dropout analysis was conducted to compare patients who completed the Klenico system and the PHQ-9 and BDI-II questionnaires at discharge with those who did not complete these assessments. The analysis examined potential differences in demographic characteristics, questionnaire scores (KDD, PHQ-9, BDI-II, and SWLS), and length of stay using 2-tailed *t* tests or Wilcoxon rank sum tests. This approach aimed to identify any systematic biases between the groups.

Post hoc power analyses were conducted to assess the statistical power of the results (using the functions *pwr.r.test*, *pwr.t.test*, and *power.rmcorr*).

### Ethical Considerations

Ethical approval was not required for the studies because according to German law, no ethical approval was required for the use of the Klenico system in routine diagnostic procedures. With reference to the Council Directive 93/42/EEC of the European Union and the according German legislation (Medizinproduktegesetz (MPG) § 23b), the test-related use of a medical device with certification (Conformité Européenne marking) does not require an ethics vote if it is carried out within the scope of its intended purpose [56]. Furthermore, the use of the Klenico system in routine diagnostic procedures falls within the scope of the responsibility of the attending physician (Berufsordnung der Ärztekammern §15). According to the ethics committee of the Ludwig Maximilian University Munich, such data collection with the aim of evaluating the performance of an accepted, evidence-based practice (quality assurance) does not require ethical approval. Finally, no ethical approval was required for analysis of the data, since retrospective studies using already available, anonymized data are exempt from needing ethics approval, according to the guidelines of the institutional review board at the Ludwig Maximilian University Munich. The study was conducted in accordance with the local legislation and institutional requirements. The participants provided their written informed consent to participate in this study. Data were anonymized. No compensation was provided.

## Results

### Reliability

Excellent internal consistency was demonstrated by the KDD both at admission (Cronbach α=0.91) and at discharge (Cronbach α=0.93).

### Exploratory Factor Analysis

A KMO value of 0.91 and a significant Bartlett test indicated eligibility for EFA. Parallel test and fit estimation favored a 7-factor solution (Multimedia Appendices 5 and 6). The 7-factor model revealed good fit (*χ*^2^_269_=382.912, comparative fit index=0.971, Tucker‒Lewis coefficient=0.950, standardized root mean square residual=0.025, and root mean square error of approximation=0.031). Based on the rotated loadings presented in Table 2, the 7 factors were interpreted and labeled as “inadequacy,” “sleep disturbance,” “affect,” “anhedonia,” “somatization,” “appetite decrease,” and “self-hatred.” The item “feelings of worthlessness” was cross-loaded onto 2 factors. Four items (“psychomotor agitation,” “psychomotor retardation,” “inner tension,” and “indecision”) did not load adequately on any factor.

**Table 2:** Exploratory factor analysis (EFA) factor structure with rotated loadings of the 31 items of the Klenico Depression Domain (KDD). All reported factor loadings are statistically significant at *P*<.05, unless otherwise specified.

	Inadequacy	Sleep disturbance	Affect	Anhedonia	Somatization	Appetite decrease	Self-hatred
Depressed mood	—^a^	—	–0.338^b^	–0.171	–0.239	—	—
Loss of interest	—	—	—	–0.229	–0.380^b^	—	—
Loss of pleasure	—	—	—	–1.111^b^	—	—	—
Loss of energy	—	—	—	–0.108	–0.723^b^	—	—
Tiredness	—	—	–0.274	—	–0.493^b^	—	—
Difficulty falling asleep	0.143	–0.350^b^	–0.135	—	–0.184	—	0.157
Disturbed sleep	—	–0.833^b^	—	—	—	—	—
Lack of restful sleep	0.220	–0.497^b^	–0.165	—	–0.221	—	—
Early morning awakening	—	–0.602^b^	—	—	—	—	—
Hypersomnia	0.163	0.147	—	—	–0.435^b^	—	—
Weight loss	—	—	—	—	—	0.455^b^	—
Loss of appetite	—	—	—	—	—	0.591^b^	—
Increased appetite	—	—	—	—	–0.416^b^	–0.241	—
Psychomotor agitation	—	—	—	—	—	—	—
Psychomotor retardation	—	—	—	—	0.150	—	—
Inner tension	—	–0.236	—	—	–0.210	—	—
Indecision	–0.264	–0.137	—	—	–0.228	—	—
Diminished ability to think	—	—	–0.194	—	–0.310^b^	—	—
Rumination	–0.233	–0.148	–0.301^b^	—	—	—	—
Feelings of hopelessness	—	—	–0.673^b^	—	—	—	—
Feelings of worthlessness	–0.395^b^	—	—	—	—	—	0.300^b^
Loss of self-esteem	–0.547^b^	—	—	—	—	—	—
Unreasonable feelings of guilt	—	—	—	—	—	—	0.816^b^
Unreasonable feelings of self-reproach	—	—	—	—	—	—	0.869^b^
Low self-esteem	–0.529^b^	—	—	—	—	—	0.184
Crying	–0.183	—	–0.263	—	—	—	0.128
Despair	—	—	–0.780^b^	—	—	—	—
Loss of affective reactivity	—	—	–0.186	–0.389^b^	—	—	—
Circadian rhythms: worse in the morning	—	–0.239	—	—	–0.210	—	—
Loss of libido	—	–0.161	—	—	–0.291	—	—
Suicidality	—	—	–0.297	—	—	—	—

^a^Not applicable.

^b^Factor loadings greater than 0.3.

### Convergent and Divergent Validity

Regarding convergent validity, the KDD showed very high correlations with both the BDI-II (*r*=0.70; *P*<.001) and PHQ-9 (*r*=0.68; *P*<.001). Regarding divergent validity, moderate associations were apparent with the PHQ-15 (*r*=0.35; *P*<.001). In contrast, the GAD-7 (*r*=0.51; *P*<.001) and the SWLS (*r*=–0.51; *P*<.001) were strongly correlated with the KDD.

### Sensitivity to Change

The KDD mean scores of the improved symptom group (n=28) demonstrated a significant difference (*t*_27_=5.36, *P*<.001) between admission (mean 36.85, SD 16.43) and discharge (mean 23.90, SD 18.54), with a high effect size (Cohen *d*=0.79) and a standardized response mean of 1.01. The means of the other measurements can be found in Multimedia Appendix 7. Values of the unchanged symptom group could not be reliably calculated due to the small group size (n=4). A total of 27 patients were excluded from the analysis due to missing BDI-II and PHQ-9 scores or deteriorated symptoms. Additionally, the KDD revealed a high repeated measures correlation with both the BDI-II (*r*=0.61; *P*<.001) and the PHQ-9 (*r*=0.59; *P*<.001). The dropout analysis revealed significant differences between patients who completed the Klenico system and standard questionnaires at discharge and those who did not. Completers showed higher scores in the KDD, BDI-II, and SWLS, and a longer length of stay (see Multimedia Appendix 7 for detailed results). Post hoc analysis revealed statistical power above 0.80.

## Discussion

### Principal Findings

This research assessed the validity of the KDD by focusing on its construct validity, reliability, and sensitivity to change.

Regarding KDDs’ underlying structure, the EFA suggested a 7-factor model (Table 2). The items on “psychomotor retardation” and “agitation” as well as for “inner tension,” “indecision,” “crying,” “circadian rhythms: worse in the morning,” “loss of libido,” and “suicidality,” did not adequately load on any of the factors.

The 7 factors of the KDD roughly reflect the 10 diagnostic criteria for depression as outlined in the classification of *ICD-10*. Table 3 summarizes which KDD factors reflect which *ICD-10* diagnostic criteria. The KDD factors “inadequacy,” “sleep disturbance,” “self-hatred,” “anhedonia,” “affect,” and “appetite decrease” each reflect a single *ICD-10* criterion. The factor “somatization” maps on 3 criteria.

**Table 3:** Overview of the International Statistical Classification of Diseases, Tenth Revision (ICD-10) diagnostic criteria reflected by the Klenico Depression Domain (KDD) factors. Overview of the ICD-10 diagnostic criteria reflected by the KDD factors.

KDD factors	*ICD-10* diagnostic criteria
Inadequacy	Loss of confidence or self-esteem
Sleep disturbance	Sleep disturbance any type
Anhedonia	Loss of interest or pleasure in activities that are normally pleasurable
Self-hatred	Unreasonable feelings of self-reproach or excessive and inappropriate guilt
Affect	Depressed mood
Somatization	Decreased energy or increased fatiguability Complaints or evidence of diminished ability to think or concentrate... Change in appetite (decrease or increase) with corresponding weight change
Appetite decrease	Change in appetite

These results indicate that the *ICD-10* criteria for depression are subject to a complex and heterogeneous structure composed of different symptom dimensions. This is underlined by the fact that the latent structure of depression is widely disputed [57]. A recent study on the factor structure of the *DSM-IV* major depression criteria showed comparable results to our findings, as it demonstrated a multidimensional symptom structure with 5 factors for the 24 criteria [58].

The multiple mappings of the “somatiziation” factor on the *ICD-10* criteria and the inadequate loading of some items can be explained by the sample-specific characteristics, the Klenico system, and the structure of depression.

The factors “somatization” and “appetite decrease” reflect those *ICD-10* criteria categorized as somatic symptoms in depression that have been assigned to a specific somatic factor in various previous studies [57]. This confirms that the heterogeneous group of somatic symptoms has an underlying common structure that is separate from the rest of the affective and cognitive symptoms of depression.

A possible explanation for the split into 2 separate somatic factors is that loss of appetite and the resulting weight loss are related to the overall severity of depression and may be determined by the severity of other symptoms, such as anhedonia, which is usually most pronounced in severely affected patients, potentially leading to a small variance and a possible floor effect [59]. Finally, the separation found between general somatic symptoms and sleep disturbance does not contradict the factorial validity of the KDD, since previous studies have shown that the 2 symptom dimensions impose separate factors [58].

Effects comparable to those at the factor level may also have occurred at the item level. The lack of significant factor loadings for “psychomotor agitation” and “retardation” may be due to their inclusion only in the clinical validation and not in the Klenico self-report module. These symptoms are more accurately assessed through external observation rather than self-report and are often more pronounced in severe depression [23,60], which can lead to reduced variability and a resulting floor effect in the data. This smaller variance might hinder their ability to align clearly with a specific factor in the analysis. The same reasoning accounts for the items for “suicidality” and “loss of libido,” both of which were just below the cutoff of 0.3—“suicidality” with loads just not on “affect” and “loss of libido” with loads just not on “somatization.” Their prevalences are correlated with the severity of depression [59,61], and acute suicidality is mainly an indication for treatment in a psychiatric clinic. Since the present sample comes from a psychotherapeutic clinic in which suicidality is an exclusion criterion, it is to be expected that such patients were less present.

“Inner tension,” “indecision,” “circadian rhythms: worse in the morning,” and “crying” did not load strongly on a single factor, instead showing distributed loadings across multiple factors. “Inner tension” aligns with both the “sleep disturbance” and “somatization” factors, indicating its role in exacerbating both sleep issues and physical symptoms. “Indecision” correlates with the “somatization” and “inadequacy” factors, highlighting its connection to cognitive impairments and reduced self-confidence typical in depressive episodes. “Circadian rhythms: worse in the morning” describes the symptom of morning mood lows in depression. Due to the wording, it is likely that this item is answered in a rather undifferentiated way and can therefore be associated by patients with both poor sleep and physical effects. “Crying” is a universal symptom that may not load strongly on any specific factor, likely due to its general association with various forms of distress across the depressive spectrum. It possibly associated with multiple factors such as “affect” and “self-hatred,” reflecting its role as a common expression of emotional pain, sadness, and feelings of low worth. This pattern suggests these items intersect various dimensions of depression, making their specific factor alignment more complex. Overall, the findings confirm that depression is a multidimensional construct and suggest that the KDD is determined by the heterogeneous structure of the *ICD-10* criteria. Since the structure of the KDD faithfully mirrors the *ICD-10* criteria, this offers initial support for its factorial validity.

Concerning convergent validity, large associations were found between the KDD and the corresponding BDI-II and PHQ-9, as expected. Additionally, the KDD showed medium associations with divergent PHQ-15. Exceptions were the GAD-7 and SWLS, with high-range correlations.

Similar to the structure of depression, the factor structure for different diagnostic tools is widely debated. The number of factors found for the BDI-II ranges between 2 and 4 [44] and for the PHQ-9 ranges between 1 and 2 [62]. For both, various compositions of the different items loading on the different factors were found. However, although the KDD reflects the factor structure according to *ICD-10* criteria, it showed significantly high associations with these 2 instruments, thus indicating the measurement of a comparable construct of depression.

The results regarding divergent validity are not surprising, as comorbidity between depression and various anxiety disorders is highly frequent [63], and depressive symptoms show structural overlap with anxiety disorders [64]. This is further supported by other studies demonstrating strong intercorrelations between the GAD-7 and PHQ-9 [65], as well as significant correlations between the GAD-7 and BDI-II. [66]

Moreover, depressive disorders are strongly negatively associated with life satisfaction and subjective well-being [67]. The strong negative correlation with the SWLS aligns with theoretical expectations and highlights the substantial impairment in life satisfaction that accompanies depressive symptomatology.

The results therefore do not counteract the construct validity of the KDD but rather support its ability to capture the broader context of psychological distress and well-being.

Regarding sensitivity to change, the KDD was able to detect improvements in symptom severity according to PHQ-9 or BDI-II sum scores and showed strong associations with changes in the BDI-II and PHQ-9 sum scores. This demonstrates that the KDD is highly sensitive to symptom change and is therefore able to monitor treatment outcomes, similar to the commonly used self-report questionnaires.

Overall, the results show that the KDD is a reliable and valid instrument for the diagnosis of depression according to *ICD-10* criteria while being able to adequately recognize improvements in symptom severity. In contrast to routine diagnostic procedures, the KDD enables a state-of-the-art diagnostic procedure that includes both self-report and clinical validation by experts and is additionally able to assign comorbidities by covering all relevant mental disorders (n=12).

A major strength of this study is that the KDD was tested in a large clinical sample under real-world conditions. It could thus be demonstrated that the KDD measures adequately in patients with frequent comorbidities in clinical reality, providing real-life evidence of its validity.

### Limitations and Outlook

A limitation of the study was the small sample size at discharge. Despite the fact that the post hoc power analysis has shown sufficient power for the *t* tests and the repeated measurement correlation, the findings regarding the sensitivity of change should be considered preliminary. Furthermore, the analysis of the sensitivity of change in the unchanged symptom group was not feasible due to the limited sample size and requires further research. In addition, the patients who used the Klenico tool at discharge exhibited greater symptom severity than those who did not. A reason for this higher representation may be that those more heavily burdened patients perceived greater relevance in completing the exit assessment, leading to higher motivation. The composition of the sample also imposed certain limitations. On the one hand, less severe cases were to be expected in the psychotherapeutic setting, and on the other hand, the design of the study could further lead to the possible exclusion of severe depression cases. This was shown by the fact that, for example, “severe depressive episode with psychotic symptoms” were extremely rare, but also “mild depressive episode” were relatively uncommon. Since the completion of the Klenico assessment requires a certain level of motivation and cognitive abilities, patients affected by severe concentration problems and anhedonia may have faced difficulties in participation. These circumstances led to certain restrictions in the factor analysis, for example, the nonloading of the item “psychomotor retardation.” Additionally, the sample exhibited a gender imbalance, with a majority (68%) being female. This mirrors the real-world scenario where women are more commonly affected by mental disorders compared with men [68]. However, this imbalance could also impact the outcome, given that different genders may exhibit variations in the manifestation of depressive symptoms [69]. Moreover, the age distribution of our participants, concentrated primarily in the younger adult segment and in the segment aged around 55 years, suggests a demographic skew that may have influenced the clinical profile observed. For these reasons, the use of the psychotherapeutic sample in this study might restrict the generalizability of the findings to broader populations, particularly individuals with severe depression. Therefore, it would be valuable to assess the functionality and generalizability of the KDD in different settings that involve patients with more severe symptoms and with a balanced gender and age distribution. This would provide a more comprehensive understanding of the measure’s effectiveness across diverse populations.

This analysis focused on examining Klenico assessment of symptom severity, and Klenico-suggested diagnoses were not examined. Therefore, this should be added in future analyses. Finally, the current validation was limited to the German version of the Klenico system. Further validation studies are necessary to assess the system in other languages, such as English. While the EFA provided initial insights into the factor structure of the KDD, these results need to be confirmed through a confirmatory factor analysis. Additionally, the factor structures of other Klenico domains also require assessment to ensure the comprehensive validity of the system across different mental health conditions.

### Conclusions

The KDD presents itself as an accurate tool for measuring depressive symptoms and a valuable tool for measuring depressive symptoms and monitoring treatment outcomes in psychotherapeutic settings. As an integral part of the Klenico system, it comprehensively covers the whole range of depression symptoms according to *ICD-10* and might therefore be a comprehensive alternative for the diagnosis and course measurement of depression. The current assessment represents an important next step in validating the psychometric properties of the Klenico system. Therefore, it may help to avoid misdiagnosis and incorrect therapies through standardized and evidence-based diagnostic procedures, thus reducing health care costs and minimizing the burden on the patients.

## Appendix

Multimedia Appendix 1 Item names, means and SDs of the different KDS items, as well as an exemplary selection of the item formulations.

Multimedia Appendix 2 Frequencies of ICD-10 diagnostic categories. Groups were formed by assigning each individual diagnosis of each patient to the corresponding disorder domain. Multiple diagnoses are possible. Listed frequencies are relative to the number of total given diagnoses. ICD-10: International Statistical Classification of Diseases, Tenth Revision.

Multimedia Appendix 3 The most frequent somatic diagnoses in the sample, in descending order.

Multimedia Appendix 4 R code for the exploratory factor analyses.

Multimedia Appendix 5 Exploratory factor analysis fit estimations for different number of factors.

Multimedia Appendix 6 Scree plot of exploratory factor analysis factor estimation.

Multimedia Appendix 7 Means, SDs, medians, and IQRs of demographic parameters and standard questionnaires at admission for the whole patient sample, patients who were measured at discharge, and patients who were not measured at discharge.

## Abbreviations

BDI-II: Beck Depression Inventory
DSM: Diagnostic and Statistical Manual of Mental Disorders
EFA: exploratory factor analyses
GAD-7: Generalized Anxiety Disorder-7
ICD-10: International Statistical Classification of Diseases, Tenth Revision
KDD: Klenico Depression Domain
KMO: Kaiser‒Meyer‒Olkin
PHQ: Patient Health Questionnaire
SWLS: Satisfaction With Life Scale

## Data Availability

The datasets generated and analyzed during this study are available from the corresponding author on reasonable request.
