# Supplementary material for: Evaluating the Construct Validity and Sensitivity to Change of the Klenico Depression Domain in Psychotherapeutic Inpatient Care: Instrument Validation Study
Source: JMIR Form Res. 2025 Jul 24;9:e50504. doi: 10.2196/50504 (PMC12332459; doi:10.2196/50504)
Supplement: Multimedia Appendix 1 [file formative_v9i1e50504_app1.pdf]

Multimedia Appendix 1

Item Name	Item Mean	Item SD	Item Formulation Self-Report (Exemplary Selection)	Item Formulation Clinical Validation (Exemplary Selection)
Depressed mood	38.70	39.51	For at least the last two weeks, I've constantly been feeling sad and depressed.	Have you been feeling constantly depressed and sad for at least the past two weeks? Is this the case for most of the day and the same on most days?
Loss of interest	37.96	35.44		
Loss of pleasure	40.01	37.20		
Loss of energy	40.58	37.09		
Tiredness	46.15	37.02		
Difficulty falling asleep	32.91	36.67	Before I fall asleep, I often lie awake for a long time (for 30 minutes at least).	Do you find it hard to fall asleep at night? Do you tend to lie awake for longer than half an hour?
Disturbed sleep	25.48	34.54		
Lack of restful sleep	44.73	38.04		
Early morning awakening	17.38	29.84		
Hypersomnia	14.92	27.75		
Weight loss	4.99	14.89		
Loss of appetite	8.63	19.98	I have no appetite.	Has your appetite diminished or disappeared completely?
Increased appetite	9.33	22.80		
Psychomotor agitation	5.93	15.41		
Psychomotor retardation	3.13	10.16		
Inner tension	53.61	33.83		
Indecision	41.74	35.45		
Diminished ability to think	29.22	36.03		
Rumination	46.82	36.82		
Feelings of hopelessness	40.27	38.27		
Feelings of worthlessness	39.22	38.88		

Loss of self-esteem	38.58	37.83		
Excessive and inappropriate guilt	30.72	32.15		
Unreasonable feelings of self-reproach	31.26	31.93	I always tell myself I've done something wrong or I've failed.	Are there things for which you have been blaming yourself lately?
Low self-esteem	39.63	35.88		
Crying	32.68	36.07		
Despair	39.35	38.54		
Loss of affective reactivity	37.54	36.50		
Circadian rhythms: worse in the morning	19.08	29.88		
Loss of libido	23.72	31.95		
Suicidality	10.77	22.30		

Multimedia Appendix 1: Item names, means and SDs of the different KDD items, as well as an exemplary selection of the item formulations.
