# Supplementary material for: Evaluating the Construct Validity and Sensitivity to Change of the Klenico Depression Domain in Psychotherapeutic Inpatient Care: Instrument Validation Study
Source: JMIR Form Res. 2025 Jul 24;9:e50504. doi: 10.2196/50504 (PMC12332459; doi:10.2196/50504)
Supplement: Multimedia Appendix 2 [file formative_v9i1e50504_app2.pdf]

Multimedia Appendix 2

Diagnostic Group	ICD-10 Code	Frequency	%
Depressive Disorders	F32.x-34.x	391	42.3
Eating disorders	F50.xx	134	14.5
Somatoform disorders	F45.xx	90	9.7
Anxiety Disorders	F40.xx-41.x	84	9.1
Reaction to severe stress, and adjustment disorders	F43.x	70	7.6
Obsessive-compulsive disorder	F42.x	56	6.1
Disorders of adult personality and behaviour	F6x.xx	50	5.4
Behavioural and emotional disorders with onset usually occurring in childhood and adolescence	F9x.xx	15	1.6
Mental and behavioural disorders due to psychoactive substance use	F1x.x	12	1.3
Dissociative (conversion) disorders	F44.xx	8	0.9
Nonorganic sleep disorder	F51.x	5	0.5
Bipolar affective disorder	F31.x	4	0.4
Disorders of psychological development	F8x.x	3	0.3
Other mental disorders due to brain damage and dysfunction and to physical disease	F06.x	1	0.1
Schizoaffective disorders	F25.x	1	0.1
Other neurotic disorders	F48.x	1	0.1

Multimedia Appendix 2: Frequencies of ICD-10 diagnostic categories. Groups were formed by assigning each individual diagnosis of each patient to the corresponding disorder domain. Multiple diagnoses are possible. Listed frequencies are relative to the number of total given diagnose
