# Supplementary material for: Evaluating the Construct Validity and Sensitivity to Change of the Klenico Depression Domain in Psychotherapeutic Inpatient Care: Instrument Validation Study
Source: JMIR Form Res. 2025 Jul 24;9:e50504. doi: 10.2196/50504 (PMC12332459; doi:10.2196/50504)
Supplement: Multimedia Appendix 3 [file formative_v9i1e50504_app3.pdf]

Multimedia Appendix 3

ICD-10 Code	Frequency
I10.90	64
M62.99	64
H93.1	52
G44.2	45
M62.89	42
M62.81	39
E03.9	38
M54.5	29
M62.88	26
E78.0	25
G43.0	25
E66.00	24
J45.0	24
M51.2	22
M53.0	22
G43.1	19
E55.9	18
E06.3	17
I10.00	17
E66.01	15
K76.0	15
M62.98	15
G47.31	14
M54.80	14
M62.90	14
G47.39	13
H91.9	13
M54.2	13
M79.70	13
R51	13

Multimedia Appendix 3: The most frequent somatic diagnoses in the sample, in descending order.
