# Supplementary material for: Evaluating the Construct Validity and Sensitivity to Change of the Klenico Depression Domain in Psychotherapeutic Inpatient Care: Instrument Validation Study
Source: JMIR Form Res. 2025 Jul 24;9:e50504. doi: 10.2196/50504 (PMC12332459; doi:10.2196/50504)
Supplement: Multimedia Appendix 4 [file formative_v9i1e50504_app4.docx]

R-code of the EFA

# Test for Multivariate Normality

mvn(data = data_CFA_extra, mvnTest="mardia", univariateTest="AD", multivariatePlot = "qq") #Not normal distributed -> use robust

estimator

#Bartlett’s Test for Sphericity and Kaiser-Meyer-Olkin (KMO) Measure of Sampling Adequacy

cortest.bartlett(data_CFA) # significant

KMO(data_CFA) #overall 0.91, all >0.5

#Unrotated Factor Analysis (1 to 7 Factors) and model comparison

fit_1f <- efaUnrotate(data_CFA, 1, estimator = "MLR")

fit_2f <- efaUnrotate(data_CFA, 2, estimator = "MLR")

fit_3f <- efaUnrotate(data_CFA, 3, estimator = "MLR")

fit_4f <- efaUnrotate(data_CFA, 4, estimator = "MLR")

fit_5f <- efaUnrotate(data_CFA, 5, estimator = "MLR")

fit_6f <- efaUnrotate(data_CFA, 6, estimator = "MLR")

fit_7f <- efaUnrotate(data_CFA, 7, estimator = "MLR")

compare <- compareFit(fit_1f, fit_2f, fit_3f, fit_4f, fit_5f, fit_6f, fit_7f, nested=FALSE)

summary(compare)

#7 factors has best fit

#Parallel Analysis

pa <- fa.parallel(data_CFA,n.iter=5000,fa='fa',plot=TRUE,SMC=TRUE,quant=0.95)

#7 factors suggested

#Rotation of Factors

rot_6f <- oblqRotate(fit_6f, method="quartimin")

summary(rot_6f, sort=F)

rot_7f <- oblqRotate(fit_7f, method="quartimin")

summary(rot_7f, sort=F)
