# Supplementary material for: Evaluating the Construct Validity and Sensitivity to Change of the Klenico Depression Domain in Psychotherapeutic Inpatient Care: Instrument Validation Study
Source: JMIR Form Res. 2025 Jul 24;9:e50504. doi: 10.2196/50504 (PMC12332459; doi:10.2196/50504)
Supplement: Multimedia Appendix 5 [file formative_v9i1e50504_app5.pdf]

Multimedia Appendix 5

Factor Number	χ2	DF	RMSEA	CFI	TLI	SRMR
7	382.912	269	0.031	0.971	0.950	0.025
6	436.28	294	0.034	0.963	0.942	0.030
5	563.847	320	0.042	0.937	0.909	0.034
4	669.838	347	0.047	0.915	0.886	0.038
3	855.027	375	0.055	0.873	0.842	0.044
2	1104.522	404	0.065	0.814	0.786	0.054
1	1379.302	434	0.074	0.738	0.720	0.065

Multimedia Appendix 5: EFA fit estimations for different number of factors. CFI = Comparative fit index, DF = Degrees of freedom, RSMEA = Root mean square error of approximation, SRMR = Standardized root mean square residual, TLI = Tucker Lewis index.
