# Supplementary figures and images for: Evaluating the Construct Validity and Sensitivity to Change of the Klenico Depression Domain in Psychotherapeutic Inpatient Care: Instrument Validation Study
Source: JMIR Form Res. 2025 Jul 24;9:e50504. doi: 10.2196/50504 (PMC12332459; doi:10.2196/50504)

## Multimedia Appendix 6

*Scree plot of EFA factor estimation*

### Parallel Analysis Scree Plots

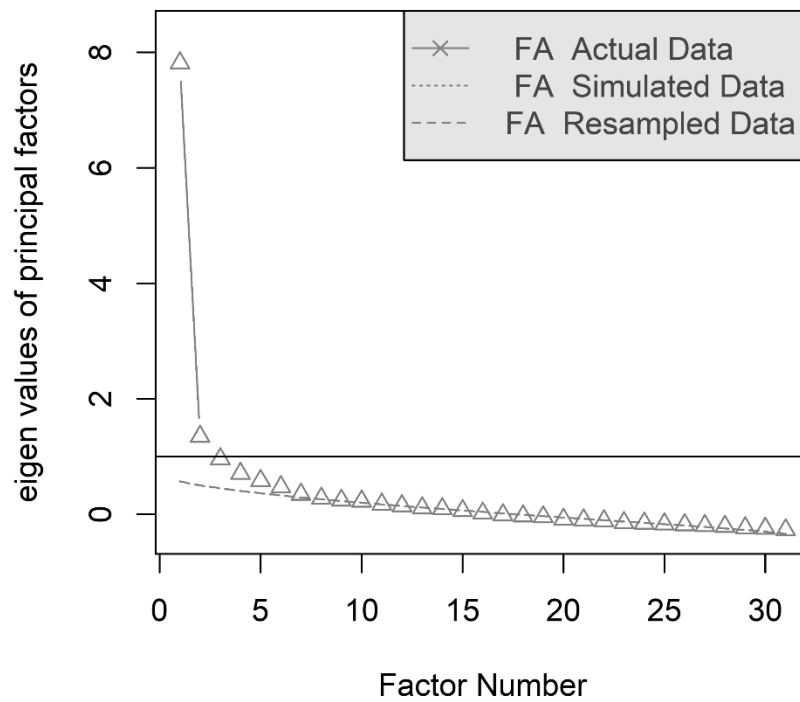

Supplement: Multimedia Appendix 6 [file formative_v9i1e50504_app6.pdf]
