# Supplementary material for: Evaluating the Construct Validity and Sensitivity to Change of the Klenico Depression Domain in Psychotherapeutic Inpatient Care: Instrument Validation Study
Source: JMIR Form Res. 2025 Jul 24;9:e50504. doi: 10.2196/50504 (PMC12332459; doi:10.2196/50504)
Supplement: Multimedia Appendix 7 [file formative_v9i1e50504_app7.pdf]

Multimedia Appendix 7

	All patients		Patients measured at discharge		Patients not measured at discharge			
	**Mean (SD)**	**Median (IQR)**	**Mean (SD)**	**Median (IQR)**	**Mean (SD)**	**Median (IQR)**	**p**	**W**
Age (years)	38.90 (14.98)	39 (29)	36.37 (15.66)	40 (28)	39.24 (14.87)	32 (29)	0.180	14278
Nr. of mental diagnoses	1.87 (1.02)	2 (1)	1.01 (1.08)	2 (1)	1.88 (1.82)	2 (1)	0.569	13186
Nr. of somatic diagnoses	3.45 (3.03)	3 (4)	3.00 (3.26)	2 (4)	3.50 (3.02)	3 (4)	0.101	14572
Length of stay (days)	70.21 (38.55)	64 (35)	102.07 (48.18)	92 (54.5)	65.91 (34.97)	58 (33)	**0.000**	**5952**
KDD (mean score)	30.02 (16.95)	29.97 (25.88)	36.08 (15.94)	38.26 (23.35)	29.20 (16.94)	28.35 (25.97)	**0.002**	**9669.5**
PHQ-9 (sum score)	14.76 (5.78)	15 (9)	15.85 (6.17)	15.5 (8.75)	14.60 (5.71)	15 (9)	0.135	8639
BDI-II (sum score)	29.26 (11.47)	29 (16)	33.18 (11.98)	35 (18.25)	28.68 (11.30)	28 (17)	**0.007**	**8260.5**
PHQ-15 (sum score)	12.57 (5.25)	12.5 (8)	13.37 (5.17)	14 (6.5)	12.45 (5.26)	12 (8)	0.175	8753.5
PHQ-GAD-7 (sum score)	11.37 (4.87)	12 (7.25)	11.59 (4.73)	11.5 (7.75)	11.33 (4.89)	12 (8)	0.760	9627
SWLS (sum score)	17.19 (6.71)	17 (11)	15.45 (5.66)	15 (6)	17.45 (6.82)	17 (11)	**0.047**	**11246**

Multimedia Appendix 7: Means, SDs, Medians, and IQRs of demographic parameters and standard questionnaires at admission for the whole patient sample, the patients who were measured at discharge and of patients who were not measured at discharge. P-value and W-value of Wilcoxon test for dropout analysis are presented. Among the measured patients, 72% were female and 33% were male. Among the not measured patients, 67% were female and 33% were male.
